# RELIABILITY OF UPPER LIMB KINEMATIC VARIABLES: ADULTS WITH UNILATERAL CEREBRAL PALSY PERFORMING A STANDARDIZED DRINKING TASK

**DOI:** 10.2340/jrm.v57.42583

**Published:** 2025-11-12

**Authors:** Camilla AKSDAL, Viven JØRGENSEN, Arve OPHEIM, Linda RENNIE

**Affiliations:** 1 Sunnaas Rehabilitation Hospital, Unit for Rehabilitation Technology, Department of Research and Innovation, Bjørnemyr; 2 Sunnaas Rehabilitation Hospital, Research Department, Bjørnemyr, Norway; 3 University of Gothenburg, Institute for Neuroscience and Physiology, Gothenburg, Sweden; 4 Oslo Metropolitan University, Faculty of Health Science, Department of Rehabilitation Science and Health Technology, Oslo, Norway

**Keywords:** muscle spasticity, cerebral palsy, task performance and analysis, kinematics, movement quality, upper extremity, functional task

## Abstract

**Objective:**

To evaluate the test–retest reliability and agreement of kinematic variables measured with an optical motion capture system during a standardized drinking task in adults with unilateral cerebral palsy (CP).

**Design:**

Test–retest.

**Subjects/patients:**

25 subjects (12 males) with spastic unilateral CP, aged 18–60 years.

**Methods:**

Kinematic variables were collected while participants performed a drinking task with the affected upper limb on 2 consecutive days. Inter-session reliability and agreement were estimated using the intra-class correlation coefficient (ICC) 2.1, and standard error of measurement (SEM).

**Results:**

Reliability was excellent for all investigated variables (ICCs: 0.90–0.99). Agreement for elbow flexion/extension, shoulder flexion, and abduction: SEM ≤ 3.1 degrees, max trunk displacement during reaching: SEM = 9.9 mm, number of stops/starts during total movement: SEM = 2.1 movement units, total movement time: SEM = 1.0 s.

**Conclusion:**

Optical motion capture of a standardized drinking task was reliable for evaluating upper limb movements in adults with spastic unilateral CP. The method may be used as an outcome measure to evaluate the effect of treatments of upper limb function, as well as to follow the function over time.

## LAY ABSTRACT

Adults with cerebral palsy (CP) experience problems with reaching, grasping, and releasing objects due to decreased motor control. Specialized camera systems, which are now clinically available, could be a step forward in objective assessment of upper limb function in neurological populations. The main aim of this study was therefore to evaluate the consistency of motion variables obtained during a drinking task using a motion-capture system. Twenty-five adults with CP were measured on 2 occasions while performing this task. The results showed a high level of consistency between the 2 measurements for all investigated motion variables. We therefore concluded that the method may be used as an outcome measure to evaluate the effect of treatments of upper limb function, as well as to follow the function over time.

Cerebral palsy (CP) is a collective term for several non-progressive, but often changing, motor impairments related to injury in the immature brain (1–3).

The ability to stretch, grasp, move, and release an object requires a high degree of coordination and timing, and these are important basic skills for performing daily activities such as eating and drinking. Many children and adults with CP have problems with the timing and the coordination of grasping and releasing objects, and therefore struggle with performing these functional tasks (4, 5).

Upper extremity function and effects of treatment are typically evaluated through functional tests such as the Action Research Arm Test (ARAT) (6) or Fugl-Meyer Assessment Upper Extremity (FMA-UE) (7), while the Modified Ashworth Scale (MAS) (8) is used to evaluate spasticity-reducing interventions (9, 10). However, there are limitations with regard to the ecological validity, reliability, and sensitivity of these tests (11). The use of optical motion capture systems for the evaluation of upper limb function in specialist healthcare could be an important step forwards, facilitating increased understanding of complex movement patterns and compensatory strategies caused by reduced motor control and muscle strength, spastic dystonia, spasticity, spastic co-contractions, and spasms. Such systems may be used to obtain objective and valid outcome variables to evaluate treatment effects (12).

Upper limb movements are highly variable and complex, often involving all degrees of freedom of all the joints in the kinematic chain when carrying out functional tasks. In goal-oriented, upper limb functional movements there is a variation in how the task is solved, due to the objective of the task, the object to be manipulated, and the context of the situation. Grasping tasks are influenced by the size and the anticipated or known weight of the object, as well as whether it is a simulated or a real object. For example, movements may be faster, and peak velocity achieved earlier during reaching when the person must pick up an empty compared with a filled cup (13). This has led to differences in test set-ups and the biomechanical models used, when evaluating arm and hand function with optical motion analysis (12).

From a clinical perspective it was important to select an examination method for upper limb movements that was both feasible to implement and capable of providing data with a sufficient degree of accuracy. The kinematic model and standardized test protocol developed by Alt Murphy et al. (13, 14) was therefore considered appropriate. This method evaluates a person’s ability to reach forward, grasp a glass of water, take a sip, and put the cup back on the table. This is a task that is both functionally relevant and ecologically valid. It requires complex coordination of multiple joints and fine motor control and is representative of a common daily activity performed across cultures and age groups. As such, it provides meaningful information concerning upper limb function in real-life contexts, addressing both the ability to perform actions and participation in everyday life. During initial validation of the model, using healthy adults, key variables within the task were determined using principal component analysis. Normative values for the task were established and the reliability of these key variables was found to be good (13). This method has since been used to evaluate upper limb function in adults post-stroke (15).

Such a common, purposeful, and functional drinking task, requiring complex inter-joint coordination skills, could also be a relevant task for the evaluation of upper limb function in adults with CP. However, the reliability of this method in this population has not yet been reported. The purpose of this study was therefore to investigate the test–retest reliability and the agreement in kinematic key variables captured during a standardized drinking task in adults with spastic unilateral CP.

## METHODS

The current study conforms to the Guidelines for Reporting Reliability and Agreement Studies (GRRAS) (16), which has been specifically developed to suit studies of reliability and agreement between measurements. The Regional Committee for Medical and Health Research Ethics in South-East Norway gave ethical approval for the study (Dnr: 2017/341). All participants gave their written informed consent.

### Participants

Adults with spastic unilateral CP admitted to Sunnaas Rehabilitation Hospital, Norway were recruited in the period January 2018 to December 2018. Twenty-five adults (12 males) were included based on the following criteria: a verified diagnosis of spastic unilateral CP, a Gross Motor Function Classification System (GMFCS) level of I–III, between 18 and 60 years of age, and with the ability to complete a drinking task with their most affected limb according to the protocol (13), and, based on this functional criterion, their manual ability corresponded to levels I–III of the Manual Ability Classification System (MACS) (21). Exclusion criteria were other diagnoses or conditions that could affect upper limb function.

### Sample size

There is, as far as we know, no established consensus on the required number of participants to be used in ICC and SEM calculations (17). One source claims that the number of participants multiplied by the number of measurements should be at least 25 in a reliability study (18). Based on earlier recommendations suggesting that 15–52 participants may be sufficient to estimate ICCs above 0.80 with reasonable precision (19), we determined that 25 participants would be acceptable for a feasibility-focused reliability study. Also, the low prevalence of unilateral cerebral palsy, the time, and practical, clinical constraints placed on the study, argued for that choice.

### Procedures

Participants underwent 2 test sessions performed at the same time of the day, on 2 consecutive days. They were instructed to refrain from any strenuous activities or physical training on the same day. In the first test session, gross motor function and upper limb function were determined using the GMFCS (20), the Manual Ability Classification System, (MACS) (21), and the ARAT (10). The spasticity in elbow flexors, pronators, wrist flexors, and shoulder internal rotators were assessed with MAS (9). For thumb and fingers, classifications according to House and Zancoli were used to describe the respective functions (19, 20). The position of the thumb in an active grip was classified from type 0–4; higher scores indicate worse function (22).

### Motion analysis

A standardized kinematic model and test protocol were used for the motion capture of the drinking task (14). Reflective markers (14 mm) were attached to the forehead, the middle of the left and right acromion process, the suprasternal notch, the lateral epicondyle of the elbow, the ulnar head, and the third metacarpophalangeal joint. A cluster of 3 markers was placed on the cup to ensure position tracking. Care was taken to ensure that the markers did not interfere with the participant’s ability to grasp the cup. To minimize systematic errors, the positions of the reflective markers were marked with a waterproof pen at the first session to facilitate the same marker placement for both recordings.

**Fig. 1:**
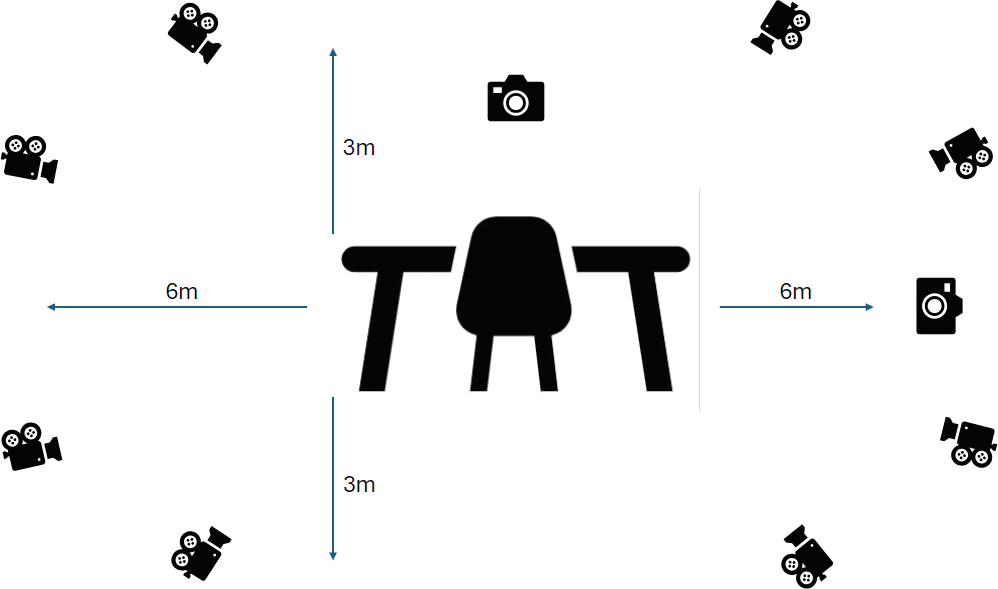
The movement laboratory is equipped with 8 infrared cameras of the Vantage type. These collect kinematic data with a frequency of 100 Hz and a margin of error of 1 mm; the cameras take up to 100 images per second. Two digital video cameras called Vue (Vicon, 2018) filmed the drinking movement in the frontal and sagittal planes.

Participants sat with hips and knees in 90 degrees of flexion, and feet positioned on the floor. The hand of the affected side was placed on the table, palm face-down, with the elbow flexed to 90° and the wrist on the table edge. They were instructed to relax the shoulder and allow the elbow to hang alongside the body. A plastic cup (height: 9 cm, diameter: 7 cm) was filled with 100 mL of water and placed on the midline of the table, 30 cm from the table edge. This distance allowed enough room for the participants to move their arm during the drinking task without necessary, compensatory trunk movements. Once in the starting position, the participants were instructed to grasp the cup, take a sip of water, and place the cup back in the starting position, at their self-selected pace. Two practice trials were performed before 5 valid trials were recorded for each session. Motion data were captured using 8 infrared cameras and 2 digital video cameras (Vicon Vantage series, Vicon Motion Capture Systems, Oxford, UK) (23). The Vicon system provides dynamic measurements down to 0.017 mm (mean) accuracy (24). Therefore, the measurement error of the infrared camera system, and its ability to detect markers accurately, is very high. An overview of the laboratory setup and camera configuration is shown in Fig. 1.

Regarding psychometric properties and measurement errors for the variables obtained during the drinking task, these have previously been assessed in patients after stroke. For patients with CP, these values have not been estimated previously, and the current study is the first to use this method in the CP population, and to evaluate the reliability of kinematic variables of the drinking task.

According to Alt Murphy (13) the drinking task in healthy people can be divided into 5 separate phases.

1. Reach – lift the hand from the starting position on the table and grab the glass.
2. Transport – bring the hand with the glass from the table to the mouth.
3. Drink – take a sip from the glass.
4. Transport – move the hand with the glass from the mouth to the table.
5. Return – release the glass and return the hand to the starting position on the table.

An overview of how the various phases were divided, the biomechanical demands, and what is needed to define a phase (13) is presented in Table I.

**Table I:** Phase definitions for drinking task

Name of phase	Start	Detected by	End	Detected by	Variable definition	Biomechanical demands
Reaching (includes grasping)	Hand movement begins	When hand velocity > 2% of max hand velocity achieved before the drinking phase, or > 20 mm/s, whichever happens first	Hand begins to move towards the mouth	First frame where velocity of the glass > 15 mm/s	Min elbow flex: Elbow angle when grasping (°) Max trunk displacement: max movement from initial position (mm)	Shoulder: Flexion Scapula: Protraction Elbow: Extension Hand and fingers: extension and supination to open hand and flexion to grasp
Forward transport (glass to mouth)	Hand begins to move towards the mouth	Frame when velocity of the glass > 15 mm/s	Drinking begins	Distance between face and glass marker <15 mm of mean distance during the middle second of the drinking phase	Max elbow flexion (°): Max elbow angle during phase Max shoulder flexion (°): Max shoulder angle during phase Max shoulder abduction (°): Max shoulder abduction angle during phase	Shoulder: Keep stable in flexed position Scapula: retraction Elbow: flexion Hand: stable in neutral position Fingers: flexed to hold the cup
Drinking	Drinking begins	Distance between the face and glass marker < 15 mm of mean distance during the middle second of the drinking phase	Drinking ends	Distance between the face and glass marker > 5 mm of the mean distance during the middle second middle of the drinking phase		Shoulder: stable in flexed position Elbow: stable in flexed position Hand: radial flexion Hand: flexed to hold the cup
Back transport (glass back to the table)	Hand begins to move towards the table	Distance between the face and glass marker > 5 mm of the mean distance during middle second of the drinking phase	Hand releases the glass on the table and begins to move back to initial position	Last frame when velocity of glass is < 5 mm/s, before the distance between the glass and hand > 20 mm		Shoulder: flexion Scapula: protraction Elbow: extension Hand and fingers: hold the cup
Return (hand back to initial position)	Hand releases the glass and begins to move back to initial position	Last frame where velocity of the glass is < 5 mm/s, before the distance between the glass and hand > 20 mm	Hand resting in initial position	Hand marker velocity returned to < 2% of maximal velocity of the returning phase, with no more frames afterwards where hand velocity > 40 mm/s		Shoulder: extension Scapula: retraction Elbow: flexion Hand and finger: extension to release and pronation to return to initial position

Table adapted from Alt Murphy (13), with additional modifications by the authors.

Vicon Nexus 2.6 (Vicon Motion Capture Systems, Oxford, UK) and a custom-written MATLAB script were used for data processing, before data were imported into MS Excel and SPSS (IBM SPSS Statistics for Windows, Version 25.0, released 2017. IBM Corp, Armonk, NY, USA) for further analysis. A detailed description of the extracted variables and their calculations is presented in Table I.

### Statistics

In this study, an intra-class correlation coefficient (ICC) 2.1 was used to report reliability, and standard error of measurement (SEM) and smallest real difference (SRD) were used to report agreement. Normality was assessed visually through Q–Q plots and histograms to ensure accurate interpretation, and also statistically using the Shapiro–Wilk test (25). Non-normally distributed variables were transformed using reciprocal and square root transformations (26).

The test–retest reliability was estimated with the intra-class correlation coefficient (ICC) with a 2-way random effects model (ICC 2.1) (27).

Level of agreement between test 1 and 2 was determined by calculating the SEM as the square root of the within-subject variance (Sw2):


$$SEM=\sqrt{S_{w}^{2}}$$ (1)

The within-subject variance was calculated as follows:


$$S_{w}^{2}=\frac{1}{n}↺\overset{n}{\underset{i=1}{\sum }}S_{w}^{2}$$ (2)

The difference between a participant’s (i) measurement and the true value is expected to be less than 1.96 SEM for 95% of the observations (27, 28). The SEM was expressed both in absolute terms and as a percentage of the group mean (SEM%). Additionally, the smallest real difference (SRD) was calculated as follows:


$$SRD=\sqrt{2}\;x\;1,96\;x\;SEM$$ (3)

Because agreement measures are quantified in the same units as the assessment tool, it lends itself to easy clinical interpretation. The SEM signifies how much an assessment score would be expected to vary if a clinician were to take the same test or measurement multiple times on the same subject. Further, both random and systematic errors are considered in the SRD score and indicate the smallest change between 2 measured values in the same individual that cannot be assigned to normal variation, but only to a true change of circumstance (28, 29).

Bland–Altman plots were used for visual interpretation of size and range of differences in measurements, as well as revealing any systematic errors and heteroscedasticity in the data (27).

## RESULTS

Demographic data together with clinical and functional test scores of participants are summarized in Table II. Classified according to House, 18 participants had type 0, 2 had type 1, 2 had type 2, 3 had type 3, and none had type 4. The position of the wrist and the ability to actively extend the fingers was grouped in 5 groups according to Zancoli from 0 to 3, where higher score indicates worse function (30). Fourteen participants were in group 0, eight in group 1, two in group 2A, one in group 2B and none in group 3. Mean and standard deviation (SD) values for all variables are summarized in Table III. No systematic bias or heteroscedasticity were seen in the investigated variables.

**Table II:** Participant characteristics

Item	*n* (%)	Mean (SD)			
Gender					
Male	12 (48)				
Female	13 (52)				
Age		33 (10)			
Affected side					
Right	15 (60)				
Left	10 (40)				
GMFCS I–V					
I	15 (60)				
II	10 (40)				
MACS I–V					
I	10 (40)				
II	15 (60)				
ARAT (0–57 points)		43 (7)			
	Modified Ashworth Scale: score (*n*)				
	0	1	1+	2	3
Elbow flexors	4	15	4	1	1
Pronators	14	3	0	3	5
Wrist flexors	8	11	4	1	1
Shoulder internal rotators	11	8	3	1	2

SD: standard deviation; GMFCS: Gross Motor Function Classification System; MACS: Manual Ability Classification System; ARAT: Action Research Arm Test.

**Table III:** Test–retest reliability of kinematic variables of a standardized drinking task for adults with spastic unilateral cerebral palsy (*n* = 25)

Variable	Mean T1 (SD)	Mean T2 (SD)	Mean difference (T1–T2)	SEM	SEM%	SRD	ICC (2.1) (95% CI)
Reaching phase							
Min elbow flexion (◦)	71.8 (8.8)	71.8 (7.6)	0.1	3.1	4.3	12.1	0.934 (0.851–0.971)
Max trunk displacement (mm)	54.7 (43.9)	57.4 (41.2)	2.7	9.9	17.6	27.3	0.972 (0.937–0.988)
Drinking phase							
Max elbow flexion (◦)	136.5 (6.5)	136.7 (5.8)	0.2	1.0	7.0	3.9	0.987 (0.970–0.994)
Max shoulder flexion (◦)	37.9 (21.5)	38.3 (20.4)	0.5	2.0	3.9	8.0	0.991 (0.979–0.996)
Max shoulder abduction (◦)	52.5 (14.5)	52.0 (14.1)	0.4	2.8	7.4	11.1	0.986 (0.977–0.995)
Total movement							
Total movement time (s)	9.4 (3.9)	8.6 (3.3)	0.4	1.0	10.7	3.9	0.938 (0.938–0.982)
Smoothness (nmu)	14.4 (8.7)	12.9 (7.6)	1.5	2.1	15.4	5.8	0.936 0.884–0.983)

T1: Test 1; T2: Test 2; SEM: standard error of measurement; SEM%: standard error of measurement as a percentage of the mean test 1 and test 2; SRD: smallest real difference; ICC: intraclass correlation coefficient; CI: confidence interval; nmu: number of movement units; SD: standard deviation.

**Fig. 2:**
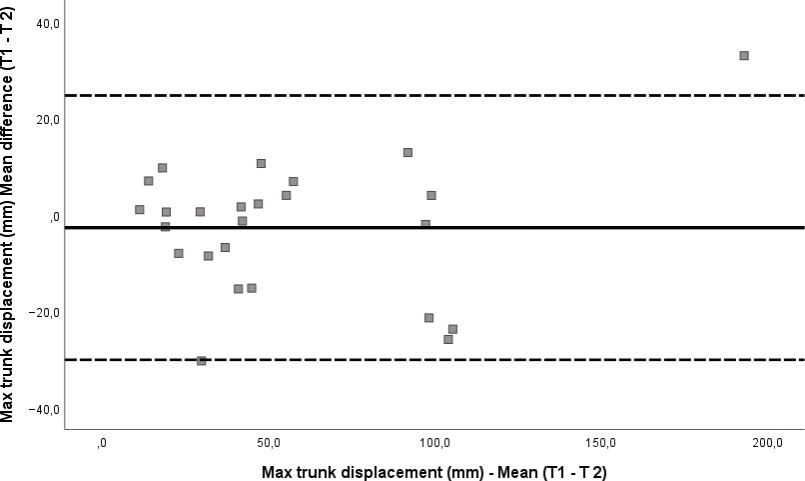
Bland–Altman plot for max trunk displacement (mm) shows calculation of the mean difference between T1 and T2. The standard deviation (SD Diff) of mean difference and the 95% limits of agreement (95% LOA) are indicated by the dashed lines.

Reliability and agreement measures for all variables are presented in Table III. ICC values for all variables were in the range from 0.93 to 0.99. The SEM and SRD estimates for the motion variables related to the upper extremity were less than 3.1° and 12.1°, respectively. This was found in both the reaching and drinking phase. Further, max trunk displacement during the reaching phase showed SEM and SRD values of 9.9 and 27.3 mm, respectively. Across the whole movement, the estimated SEM and SRD for movement smoothness was 2.1 and 5.8 movement units, respectively. For the total movement time, the SEM and SRD was 1.0 and 3.9 s, respectively. The agreement in the variable max trunk displacement is illustrated with a Bland–Altman plot (Fig. 2).

## DISCUSSION

There is a lack of reliable and standardized test protocols for motion analysis of functional upper limb tasks for adults with CP. The aim of this study was therefore to establish levels of agreement and reliability for motion-capture data of a standardized drinking task in adults with spastic unilateral CP. The main finding was good agreement and excellent reliability for all variables relative to the reaching and drinking phase of the task, as well as for the variables related to the total movement.

Previous studies investigating reliability and agreement in upper limb kinematic variables for individuals with CP have examined slightly different motion patterns such as simulated eating movements (31), hand to head, or hand to mouth movements (32), as well as a typing task (33). Further, some of these studies investigated reliability in children and adolescents with unilateral CP (4, 34–36), and one used a different statistical approach to the study (32, 37). However, despite methodological differences, these investigations reported in general good to excellent reliability (ICC ranging from 0.6–0.9) for investigated variables, thereby supporting the findings of the current study.

Interestingly, Schneiberg et al (31) studied reliability in many of the same variables as the current study, such as smoothness, trunk displacement, minimum elbow flexion, and shoulder flexion, during simulated eating in children with CP and reported high ICC values for all variables (0.82–0.95), strongly mirroring our results. Further, Mackey et al. (32) analysed bringing hand to head, as well as hand to mouth in children with CP, and, especially relevant for this study, the hand to mouth movement mirrors the forward transport and the drinking phase. They reported an elevated level of similarity in kinematic curves for elbow and shoulder flexion/extension, supporting the high reliability and level of agreement found for the same variables as in the current study. This could indicate that variation in these movements does not increase with age, and can be reliably measured in both children and adults with spastic unilateral CP. Further, it is important to consider that these studies used a simulated task, whereas the current study used a functional task. This further suggests that level of measurement error does not increase when using a higher demanding version of the task.

Also of interest is the finding that the minimum elbow flexion, maximal trunk displacement, total movement time, and movement smoothness were the most deviant variables compared with healthy adults (14). These variables reflect key aspects of impaired motor control, such as compensatory strategies and reduced efficiency, and may be particularly sensitive to clinical change.

Relevant to our results was the finding that 3 participants had values that were outliers. One was for movement time and smoothness measurement, and 2 were for trunk displacement, shoulder abduction, and shoulder flexion. These participants had among the highest MAS and lowest ARAT scores in the study group, suggesting lower arm function as well as increased compensatory strategies. The outlying values were similar over the 2 test occasions, and therefore did not affect the level of agreement determined for these variables. However, the outliers may have contributed to higher ICC values due to increased between-subject variability relative to the within-subject variability, which positively influences ICC levels (38). Therefore, all ICC values were re-examined without the outliers, showing that all variables still had ICC values above 0.90. This implies that even though the sample became more homogeneous, the degree of measurement error (within-subject variation) was so low that the participants’ measurements could still be distinguished from each other.

The 95% limits of agreement (LOA) for trunk displacement appear acceptable for clinical use, as they are within the expected range for adults with unilateral CP. Changes exceeding 27 mm are likely observable during functional tasks. Given that normative values are around 26.7 mm (14), reductions towards this range may indicate real improvement.

The smallest real difference (SRD) provides a threshold for detecting change beyond measurement error. For example, the SRD for trunk displacement was 27.3 mm. A reduction from 55 mm to 25 mm would exceed this and suggest a meaningful gain in postural control.

The SEM values, which reflect the expected measurement error of a single test occasion, were generally low for the most functionally relevant variables. This supports the precision of the test and its suitability for individual monitoring.

Changes in elbow extension, movement smoothness, and total movement time were also within clinically interpretable SRD ranges and relate directly to reaching, grasping, and functional efficiency. These measures can help therapists detect meaningful improvements and guide rehabilitation planning.

Relative reliability (ICC) is useful for group comparisons and ensuring consistency, while absolute indices like SEM, SRD, and LOA are essential for identifying true change at the individual level. Together, these findings support both discriminative and evaluative use of the test protocol in clinical settings.

### Limitations

Some potential limitations of this study should be acknowledged. An important limitation of this study is the relatively small sample size. While reliability studies ideally include a larger number of participants to allow for narrower confidence intervals around ICC estimates, recruitment was constrained by the low prevalence of adults with unilateral cerebral palsy and the need to integrate the study procedures within a clinical setting without disrupting ongoing rehabilitation. Based on prior literature suggesting that 15–52 participants may be sufficient to estimate ICCs above 0.80 with acceptable precision, a sample size of 25 was considered feasible and methodologically acceptable for the purposes of this study. Nevertheless, we acknowledge that a larger sample would have strengthened the statistical precision and generalizability of the findings. Future studies should aim to replicate these results in larger and more heterogeneous populations to further validate the method.

Another factor limiting the generalizability of the results relates to the functional level of the participants. A relatively substantial proportion of the participants had a high functional level with a GMFCS I (60%) and MACS I (40%), which limits the generalization of our results to a larger proportion of the individuals with CP. It is further worth considering that the agreement and reliability estimates are only relevant when using similar test set-ups.

Another limitation is that the kinematic model used does not measure all relevant movements during the task. Examples of this are finger and thumb motions. Thumb mobility is often affected in CP and can contribute to difficulty with grasping the cup. It would be ideal to have a model capable of capturing thumb movement, pronation and supination in the forearm, or radial/ulnar deviation in the wrist, as well as head and trunk rotation. The current procedure did not restrict trunk motion during the drinking task, allowing compensatory strategies such as forward leaning and trunk rotation to occur. However, these limitations also make this kinematic model fast and easy to use in a clinical setting. Although the use of optical motion capture is resource-intensive and requires technical expertise, it provides precise and reliable data on upper limb kinematics. This approach is not intended for routine clinical use but may be of particular value in settings that already have access to motion-analysis laboratories, such as those used for gait analysis. In such contexts, applying similar methodology to upper limb assessment may offer additional insight in selected patient populations**.**

Further, participants did not use their regular hand and/or finger orthosis during testing, which for some could have influenced their ability to grasp the cup. Future studies should consider testing both with and without the use of hand/finger orthosis to further examine the clinical applicability. Also, the testing of the least affected upper limb, relative to the affected side, could have been considered. In our clinical experience, the affected arm is used less and often has a support and relieve function for the least affected hand/arm during execution of complex tasks in daily life by adults with CP. Comparing the least and the most affected upper limb would give further insight into movement control of the arm and hand in persons with spastic unilateral CP.

To conclude, we found optical motion capture to be a reliable method for evaluating upper limb function using a standardised drinking task in adults with spastic unilateral CP. Future studies should explore if there is an added value in testing both the affected and unaffected upper limb. The kinematic model and test set-up should also be validated for other functional upper limb tasks, and with other patient groups.
